# Optimal knockout strategies in genome-scale metabolic networks using particle swarm optimization

**DOI:** 10.1186/s12859-017-1483-5

**Published:** 2017-02-01

**Authors:** Govind Nair, Christian Jungreuthmayer, Jürgen Zanghellini

**Affiliations:** 1 Department of Biotechnology, University of Natural Resources and Life Sciences, Muthgasse 11, Vienna, 1190 Austria; 2 Austrian Centre of Industrial Biotechnology, Muthgasse 11, Vienna, 1190 Austria; 3 TGM - Technologisches Gewerbemuseum, Wexstraße 19-23, Vienna, 1200 Austria

**Keywords:** Systems biology, Metabolic networks, Dual metabolic network, Minimal cut sets, Strain optimization, Knockouts, Metabolic pathway analysis

## Abstract

**Background:**

Knockout strategies, particularly the concept of constrained minimal cut sets (cMCSs), are an important part of the arsenal of tools used in manipulating metabolic networks. Given a specific design, cMCSs can be calculated even in genome-scale networks. We would however like to find not only the optimal intervention strategy for a given design but the best possible design too. Our solution (PSOMCS) is to use particle swarm optimization (PSO) along with the direct calculation of cMCSs from the stoichiometric matrix to obtain optimal designs satisfying multiple objectives.

**Results:**

To illustrate the working of PSOMCS, we apply it to a toy network. Next we show its superiority by comparing its performance against other comparable methods on a medium sized *E. coli* core metabolic network. PSOMCS not only finds solutions comparable to previously published results but also it is orders of magnitude faster. Finally, we use PSOMCS to predict knockouts satisfying multiple objectives in a genome-scale metabolic model of *E. coli* and compare it with OptKnock and RobustKnock.

**Conclusions:**

PSOMCS finds competitive knockout strategies and designs compared to other current methods and is in some cases significantly faster. It can be used in identifying knockouts which will force optimal desired behaviors in large and genome scale metabolic networks. It will be even more useful as larger metabolic models of industrially relevant organisms become available.

**Electronic supplementary material:**

The online version of this article (doi:10.1186/s12859-017-1483-5) contains supplementary material, which is available to authorized users.

## Background

Metabolic engineering aims to improve product yields in cellular systems by applying a variety of tools. Constraint based methods which use only the stoichiometry of metabolic reactions have been particularly successful in the development of strategies towards fulfilling this aim [1]. One important application is the prediction of knockouts to enforce desired metabolic behaviors in an organism. A method that allows one to predict efficient intervention strategies using the concept of minimal cut sets **MCSs**, was developed by Klamt and Gilles [2]. This was generalized to constrained minimal cut sets **cMCS**, where in addition to blocking undesired fluxes, survival of some desired fluxes is possible [3, 4]. The automatic partitioning method **APM** uses an objective function to specify the design objectives and the partitioning of fluxes into desired/undesired is done automatically to find successively larger cMCS till a global optimum is reached [5]. Previously we showed that a genetic algorithm could reach the global optimum faster than than APM [6]. However, all these methods are applicable only to small and medium-scale metabolic networks.

In a recent work by Ballerstein et al., it was shown that cMCS can be directly calculated from the stoichiometric matrix [7]. Using this method, it is possible to calculate intervention strategies even in genome-scale metabolic networks [8]. Another work extended this concept to include regulation [9]. A limitation of this method is that the desired flux or flux ratio of a metabolite has to be manually specified to get corresponding cMCS.

There exist other constraint based methods for predicting intervention strategies. OptKnock solves a bi-level optimization problem, to predict knockouts leading to maximal product formation at maximal growth [10]. A three-level optimization problem is used to maximize minimal product formation in RobustKnock [11]. OptGene uses a genetic algorithm to predict knockouts [12]. Similarly, evolutionary algorithms and simulated annealing have been used in [13]. Another metaheuristic approach was using a hybrid of bees algorithm with flux balance analysis **FBA** [14]. While these methods optimize for design goals, doing so with a minimal number of knockouts is not necessarily guaranteed.

From an engineering perspective, we would like the organism to have a guaranteed high yield for the product of interest. Given that even in the face of genetic perturbations microorganisms redirect metabolic flux towards maximizing cellular growth [15], this high yield must be maintained at high growth rates. Additionally, the number of knockouts should be as small as possible to facilitate easy implementation in the laboratory.

Here we present a new method, PSOMCS, which uses particle swarm optimization **PSO** along with the method developed in [7–9] to calculate cMCS while overcoming the mentioned limitations of other methods. Our basic motivation is to combine the computational rigour of cMCS with the flexibility of the optimization-based approaches in order to solve (non-linear) intervention problems efficiently. We aim to find not only the optimal intervention strategy for a given design but also the best possible design. In addition, we show that PSOMCS is also faster than other methods which try to find cMCS leading to optimal design objectives.

## Methods

### Calculating cMCS

A metabolic network of *m* internal metabolites connected by *n* reactions in steady state is represented by the set of linear equations

1 $$ \mathbf{Nr} = \mathbf{0}  $$

where **N** is a *m*×*n* matrix consisting of stoichiometric coefficients of all participating reactions such that each column represents one reaction. **r** is a vector of reaction fluxes. Reactions can be both reversible (*Rev*) and irreversible (*Irrev*), thereby imposing the constraint

2 $$ \mathit{r_{i}} \geq 0~\forall~ i \in {Irrev}.  $$

(1) and (2) define a flux space. Depending on the desired outcome, an intervention problem can be set up dividing this space into desired and undesired fluxes. The set of undesired fluxes for *t* reactions can be defined by

3 $$ \mathbf{Tr} \leq \mathbf{t}  $$

where $\mathbf {T} \in \mathbb {R}^{t \times n}$ and $\mathbf {t} \in \mathbb {R}^{t \times 1}$. Likewise, the set of desired fluxes for *d* reactions can be defined by

4 $$ \mathbf{Dr} \leq \mathbf{d}  $$

with $\mathbf {D} \in \mathbb {R}^{d \times n}$ and $\mathbf {d} \in \mathbb {R}^{d \times 1}$.

In [8], cMCS are calculated by first solving a series of mixed integer linear programming **MILP** problems representing (1) and (3) and then filtering those solutions which also satisfy (4). In [9], this is combined into a single system represented as (cf. equation (5) in [9])

5 $$ \begin{aligned} &\left(\begin{array}{ccccc} \mathbf{N}^{T}_{rev} & \mathbf{I}_{rev} & -\mathbf{I}_{rev} & \mathbf{T}_{rev}^{T} & 0 \\ \mathbf{N}^{T}_{irr} & \mathbf{I}_{irr} & -\mathbf{I}_{irr} & \mathbf{T}_{irr}^{T} & 0 \\ 0 & 0 & 0 & 0 & \mathbf{N} \\ 0 & 0 & 0 & 0 & \mathbf{D} \\ \end{array} \right) \times \left(\begin{array}{cc} \mathbf{u} \\ \mathbf{vp} \\ \mathbf{vn} \\ \mathbf{w} \\ \mathbf{r} \\ \end{array} \right) \begin{array}{cc} = \\ \geq \\ = \\ \leq \\ \end{array} \left(\begin{array}{cc} 0 \\ 0 \\ 0 \\ \mathbf{d} \\ \end{array} \right) \\ &\qquad\qquad\qquad\qquad\mathbf{t}^{T}\mathbf{w} \leq -c \\ &\mathbf{u} \in \mathbb{R}^{m}, \mathbf{vp},\mathbf{vn} \in \mathbb{R}^{n}, \mathbf{d} \in \mathbb{R}^{d}, \mathbf{vp},\mathbf{vn},\mathbf{w},\mathbf{r}_{\text{irr}} \geq 0, c > 0. \end{aligned}  $$

Note that the **N** and **T** matrices have been split into reversible (subscript *rev*) and irreversible submatrices (subscript *irr*). Similarly, identity submatrices for reversible and irreversible reactions are represented by the matrices **I**_*rev*_ and **I**_*irr*_ respectively. cMCS are directly calculated by finding solutions with minimum number of non-zero entries in **v** **p**,**v** **n**. Additionally binary *indicator* variables **z** **p** and **z** **n** are introduced such that *z* *p*_*i*_=0 if *v* *p*_*i*_=0 and *z* *p*_*i*_=1 if *v* *p*_*i*_>0 and similarly for *zn*, *vn*. Only one direction of **v** (either *v* *p*_*i*_ or *v* *n*_*i*_) can be active, hence

6 $$ zp_{i} + zn_{i} \leq 1.  $$

We set up the following optimization problem

7 $$ \begin{array}{c} \text{minimize} \sum_{i = 1}^{n} (zp_{i} + zn_{i}) \\ \text{s.t.}~(5), (6) \end{array}  $$

with the additional constraint that the flux through a reaction is turned off if it is part of a cMCS, i.e., *r*_*i*_=0 if *z* *p*_*i*_=1 || *z* *n*_*i*_=1.

With this system it is possible to find cMCS which will result in designs satisfying constraints on yields/fluxes specified by (3), (4). However, we would like to have a method which given some design objectives (e.g., high product yield even at high growth rates) calculates cMCS corresponding to optimal values for the design objectives. Since any design can be represented as a function of **T**,**D**,**t** and **d**, the optimization problem can be stated as

8 $$ \begin{array}{c} \text{max} ~f(\mathbf{T},\mathbf{D},\mathbf{t},\mathbf{d}) \\ \text{s.t.} (7). \end{array}  $$

In other words, the problem is to find optimal combinations of {target/desired} yields for all reactions to be optimized. This is not easy for a few reasons. In general, this is a non-linear optimization problem. Non-linear optimization is known to be inherently complex with general deterministic solutions being impossible to find. Secondly, slight adjustments in (3), (4) could result in completely different cMCS with different cardinalities. Finally, not all such combinations will result in cMCS. These issues become acute when the search space is more dense with many possible combinations, as in large and genome-scale metabolic networks. We attack this problem using PSO as it has been successfully used to find solutions to complex non-linear optimization problems in other fields [16–18].

### Particle swarm optimization

PSO is a metaheuristic inspired by the flocking behavior of birds [19]. In PSO, particles distributed within a multidimensional space collectively move towards an optimum guided by a fitness function. Particle fitness is determined by its position in the search space. The motion of a particle is influenced by its neighbours and the currently known fittest particle. More information on PSO can be found in [16–18, 20].

**Fig. 1:**
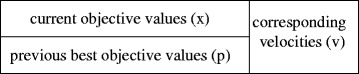
Schematic of the PSO particle. A particle stores three types of information: the current values, values corresponding to its own previous best fitness and velocities corresponding to each objective

Typically, a particle is made up of three *j*-dimensional vectors, where *j* is the dimensionality of the search space. These represent the current position *x*, its previous best position *p* which is the position corresponding to the highest fitness achieved by the particle and the velocity *v*, Fig. 1. Particle motion is guided by the following equations,

9 $${} v_{i} (t + 1) = \chi \{ v_{i} (t) + \varphi_{1} \beta_{1} [p_{i} (t) - x_{i} (t)] + \varphi_{2} \beta_{2} \: [g_{i} (t) - x_{i} (t)] \}  $$

10 $$ x_{i} (t + 1) = x_{i} (t) + v_{i} (t + 1)  $$

$$i \in \{ 1.. j \}. $$

*g* is the position corresponding to the global best fitness of the entire swarm till the current *t*. *φ*_1,2_ are called “acceleration constants” and determine the relative influence of the particle’s own knowledge and that of the group, both of which are commonly set to 2 [18, 20]. *β*_1,2_ are uniformly generated random numbers within the range (0,1] for each *i*,*t*. *χ* is the constriction coefficient first introduced in [21] and generally has a value of 0.7298 in the literature [16, 18]. This dampens the dynamics of the particles, preventing the velocity from rapidly increasing beyond the problem bounds. The amount of information available to a particle depends on its access to information of other particles. Access to a limited number of other particles is closer to the behaviour of natural swarms. In our implementation each particle is connected to four other particles, which has a comparatively better performance than other choices [22]. Additionally, we borrow a concept from [23], where in addition to its fixed neighbours, a particle also establishes connection with another randomly selected particle.

**Fig. 2:**
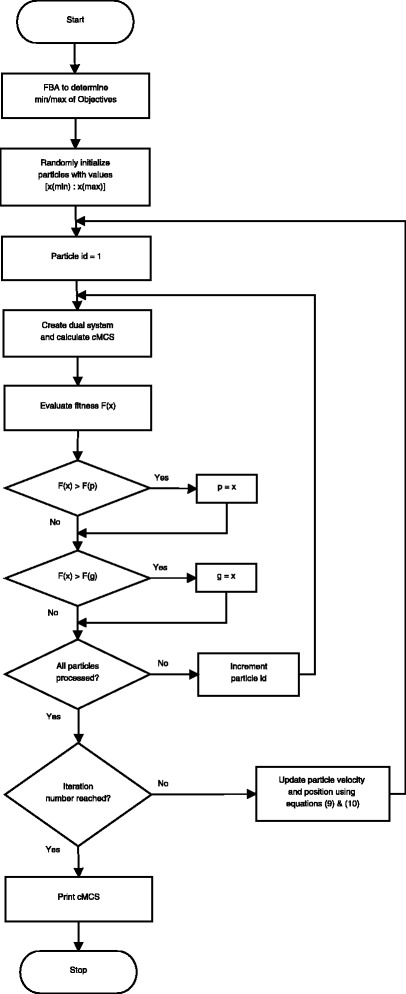
Flowchart of PSOMCS. p and g are the current particle best and global best respectively. The algorithm stops when the number of iterations reaches a pre-specified maximum or if the maximum fitness remains unchanged for a pre-specified number of iterations

The MILP given by (7) needs constraints specified by (3), (4) to calculate corresponding cMCS. For example, consider a network which has, among other reactions, a substrate uptake reaction *R*_*S*_, a reaction for the product secretion *R*_*P*_ and one for biomass *R*_*Bio*_. An optimal design could be stated as having *R*_*P*_/*R*_*S*_≥*x*_1_ and also that biomass fluxes of *R*_*Bio*_/*R*_*S*_≥*x*_2_ exist. However, we don’t know the combinations of *x*_1_, *x*_2_ resulting in optimal design. This is where a PSO can be useful. After initializing **x** **,** **v**, the set of positions and velocities for all particles, within the range of values for {*x*_1_,*x*_2_} on some constant *R*_*S*_, the PSO iteratively finds increasingly better solutions for (8) using (9) and (10) and moves towards the global optimum. The PSOMCS flowchart is shown in Fig. 2.

The fitness function will depend on the nature of the desired optimum. Considering that our objective is to have a design with high yields and minimal knockouts, the following fitness function was used,

11 $$ F(x) = \left(1 - \frac{|cMCS|}{n} \right) \cdot \prod\limits_{i} \frac{x_{i}}{x_{i}(max)}.  $$

## Results

To clarify the working of PSOMCS, we first apply our method to a small toy network, optimizing for only a single reaction. Next, to confirm the accuracy of our predictions, we compare our method against another method based on a genetic algorithm (GAMCS) which we had previously developed [6]. The model used is the medium-scale *E. coli* core model presented in [24]. Finally we find optimal intervention strategies for maximizing the minimal product yield in a genome-scale metabolic network. FBA was used to calculate the range of yields [min:max] for each objective and particles were initialised within this range. Only one solution is calculated for a MILP. The parameters used are shown in Table 1. Implementation of PSOMCS was done using Perl http://www.perl.org/. For the performance critical parts of the program, i.e., solving the MILP and also the LP, the IBM ILOG CPLEX Optimization Studio - a commercial optimization package - was used through the Math::CPLEX Perl module. Also, our algorithm is designed to make use of modern CPU architectures and can be run in parallel on multiple cores.

**Table 1:** PSOMCS parameters

Model	No: particles	No: iterations
toy network	4	2
*E. coli* core	10	40
iAF1260	10	40

Details of parameters used for the different models

**Fig. 3:**
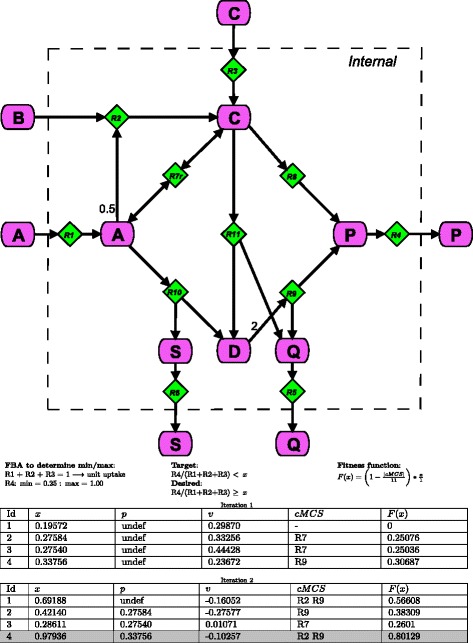
PSOMCS small example. Running the PSOMCS on a toy network. This network has three input reactions, which can be assumed to be substrates and three secretion reactions, which can be assumed to be three different products. We want to maximise the yield of R4, that is maximize (R4/(R1 + R2 + R3)). Note that the particles operate in a single dimensional search space and *x* represents the yield for R4. After performing FBA to determine the maximum and minimum yields for R4 given unit substrate uptake, four particles are initialised within this range. Initial velocities are also assigned. cMCSs are calculated after creating and solving the dual system. Fitness is a function of *x* and the cardinality of the cMCS. *g* corresponds to *x* with the highest fitness which is particle 4 after both the first and second iterations. After the first iteration, every particle except the first has a value for *p*. Note that for particle 4 a yield higher than 0.98 is guaranteed. In reality, the minimal yield with the corresponding cMCS is 1, which is also the case for particle 1. This is the value the algorithm will return if allowed to run for a few more iterations

Consider the network given in Fig. 3. We wish to find minimal knockouts which will ensure the highest possible yield for reaction R4. In the first iteration, cMCS corresponding to low yields are found. In the second iteration, all particles move towards higher yields. One particle, on the solution of its dual system gives the cMCS of ‘R2 R9’. Removal of R2 and R9 from the network blocks all flux through R5 and R6, thus redirecting the network flux through R4. This corresponds to the highest minimal yield of 1 for R4.

**Fig. 4:**
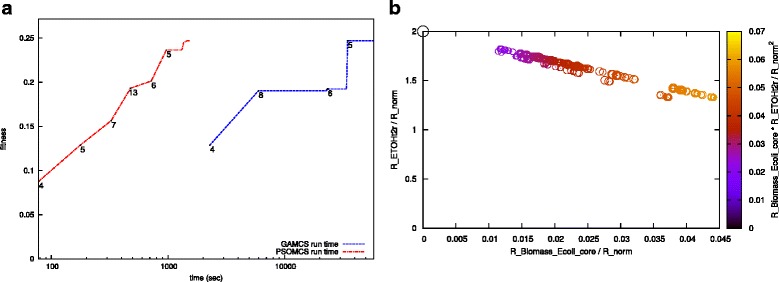
Comparing the runtimes of PSOMCS and GAMCS. **a** Plotting the runtimes of PSOMCS and the GA we had previously implemented clearly shows PSOMCS is orders of magnitude faster than GAMCS. Note that the time axis is logarithmic and that both algorithms reach the same maximum fitness. The change in knockout sizes are indecated by the numbers along the line. **b** Both methods also produce similar designs, an example of which is shown. This design is obtained with 5 knockouts (R_GND R_SUCOAS R_MALS R_ACt2r R_LDH_D). The plot was generated by applying the knockouts on the complete set of 429275 EFMs of the *Escherichia coli* core model. R_norm is the sum of uptake rates for the five carbon substrates, glucose, galactose, mannose, arabinose and xylose under aerobic conditions

We apply PSOMCS to generate designs in an *E. coli* core network which will ensure high yield of ethanol even in the face of high growth. This network was previously used to design a high yield ethanol producing strain in [24]. This model has 71 reactions and 68 metabolites. We had previously used this model to predict optimal intervention strategies using a genetic algorithm (GAMCS), which we had shown to be faster than other current approaches [6], particularly compared to APM, which is guaranteed to find the optimal solution [5]. Here we compare our approach with GAMCS in terms of speed and accuracy of results. The machine used had the following specifications − 2 CPUs, 12 cores, Intel Xeon X5650 2.67 GHz, running an Ubuntu 14.4 LTS operating system. The time taken for a typical PSOMCS and GAMCS run is plotted in Fig. 4 a. The superiority of our method in terms of speed can be clearly observed. GAMCS takes 34,857 seconds to reach the maximum fitness. PSOMCS takes only 1493 seconds for the same. This is an over 23 fold improvement in performance. In comparison, APM would not only require that the desired EFMs be assigned weights, but also the time taken by it would have been outside the boundaries of this plot. The cMCS corresponding to the optimum obtained by both GAMCS and PSOMCS are exactly the same. Figure 4 b is one of the designs corresponding to a high fitness. This design was in the solution pool of both the PSO and GA methods. In this design, a minimum ethanol yield of 1.33 is guaranteed even when the growth rate is 0.044. Also, as can be expected, production of competing by-products: acetate, lactate and succinate is blocked. Additionally, flux through the oxidative part of the pentose phosphate pathway is blocked and so is the pyruvate-malate cycling. Multiple cMCS resulting in similar design characteristics were returned by our method.

**Fig. 5:**
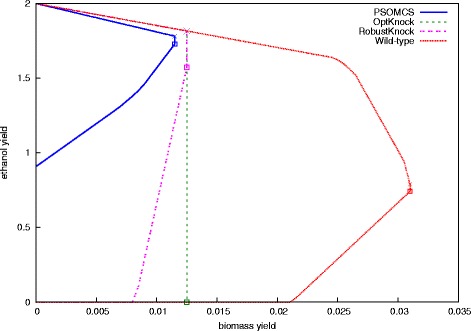
Design for a genome-scale *E. coli* model. *E. coli* was designed for enhanced ethanol production using the genome-scale iAF1260 model. For comparison, designs obtained using OptKnock and RobustKnock are also presented. The design using PSOMCS guarantees a minimal ethanol yield of 0.9, in contrast this is 0 for both RobustKnock and OptKnock. All designs have a maximum biomass production rate greater than 0.01 with the one for PSOMCS being comparatively lower. The maximum yield for all the designs is 2. The given plots have been generated by using FVA on the iAF1260 model while considering the respective knockouts produced by each method. The FBA solution space at maximum growth is highlighted, with crosses indicating the maximum and squares the minimum ethanol yield. All designs involve 5 knockouts - (R_ACALD R_GLUDy R_Htex R_PGI R_TKT2) for PSOMCS, (R_ACALD R_H2tex R_PHEt2rpp R_PPKr R_TYRtex) for OptKnock and (R_ACKr R_F6PA R_FBA R_GLCptspp R_PGCD) for RobustKnock

To test the capabilities of our method we applied it to the genome-scale model of *E. coli* presented in [25]. Our aim was to find cMCS that result in an scenario of growth-coupled ethanol yield. A few strategies were used in [8, 9] to reduce the network size. These strategies are aimed at reducing the network size and improving computational efficiency, which takes real growth conditions into account and removing all superfluous components. First, the network was reduced to grow anaerobically on glucose as the only carbon source. The resulting network has 1413 reactions and 971 metabolites. Network compression was done by combining reactions operating at fixed ratios into reaction subsets. Exchange reactions, spontaneous reactions and reactions essential for the ethanol and biomass production were excluded from participating in cMCS by setting their corresponding *zp*, *zn* variables to zero. The machine we used for this test had 24 CPUs, 396GB RAM, Intel Xeon E5-2667 2.90 GHz processor, running on Ubuntu 14.4 LTS. The cMCS cardinality was limited to 5. With 4 particles being processed in parallel, the program was run for 40 iterations. It took 14 iterations (∼ 74 hours) to find the optimal design. One of the designs is shown in Fig. 5 along with designs obtained using OptKnock and RobustKnock on the same machine. The envelope of the strain specific phenotypic solution space was calculated with flux variability analysis **FVA** [26] of the iAF1260 network while considering the respective knockouts predicted by each method. The minimally required biomass production was set at 0.006 and both were limited by unit glucose uptake and a maximum knockout size of 5. OptKnock took 4 minutes to run while RobustKnock ran for 71 minutes. The minimal ethanol yields were 0 in both cases. As can be observed, PSOMCS offers a better design with the ethanol production being strongly coupled to biomass production and at no point falls below a yield of 0.9.

## Discussion

Here we have presented a method, PSOMCS, to design strains with high minimal product yield using knockouts of minimal possible size. To do this, we employ a PSO together with the direct enumeration of cMCS developed in [7–9]. This method has made it possible to find cMCS in large and genome-scale networks. However, it is not designed to optimize engineering goals. That is, we we would like to find not only the optimal intervention strategy for a given design but the best possible design too. Finding intervention strategies that achieve this is an important goal of metabolic engineering, especially in the production of industrially important chemicals. We deliver on this goal by using a PSO built on top of the base provided by the direct enumeration of cMCS. Our method thus expands the utility of this method. Additionally we would like to point out that in the case of optimizing for a single reaction, solving (5) with continuous values within the [min:max] range for that reaction would suffice. However, in the presence of multiple objectives this task becomes computationally exhaustive and infeasible, thereby justifying the use of a metaheuristic approach such as the one used here.

There have been other methods with a similar strategy as ours, which is the use of a metaheuristic in combination with another method like linear programming. Most methods have relied on genetic algorithms [6, 12, 27], evolutionary algorithms and simulated annealing [13] and also an artificial bees algorithm [14]. Ours is the first attempt at using the dual method in a similar fashion, along with the use of a PSO.

As shown by the comparison with OptKnock and RobustKnock in Fig. 5, although all designs have the same highest ethanol yield of 2, PSOMCS provides a design with the highest guaranteed minimal ethanol yield. RobustKnock was developed to overcome the ’too-optimistic’ nature of OptKnock and this is reflected in the nature of their respective designs. Also of note is the fact that both OptKnock and RobustKnock need a minimal level of biomass production to be manually specified while PSOMCS does not. In fact, if we reduce the minimal biomass production requirement to 0.001 (in order to mimic the PSOMCS settings), RobustKnock runs for over 90 hours without finding the optimum. Running OptKnock and RobustKnock multiple times with different biomass levels will result in different solutions, some of which will be better than others. PSOMCS eliminates this need to manually set reaction fluxes and searches the entire feasible space of biomass yields to find the optimal one. Growth-coupling is a key principle in metabolic engineering. It requires that growth should only be feasible if a desired compound, like ethanol, is mandatorily produced as by-product. It can be seen in Fig. 5 that PSOMCS achieves this with a growth rate about one third of the wild-type. However, growth-coupling does not enforce nor require that the maximal product yield is attained at a non-zero growth rate. In fact Fig. 5 illustrates the rule rather than the exception, as typically the maximum product yield is achieved at zero growth [28, 29]. Furthermore, an ideal production state will be characterized by zero growth, where all available resources are used for product formation. In this senses, biomass production can be seen as an “unwanted” by-product. Recent advances in fermentation processes employ zero-growth approaches [30, 31]. However, these approaches are associated with many challenges which go far beyond the scope of the presented work. Nevertheless, Fig. 5 indicates that the presented designs retain their wild-type behavior to be operated as optimal zero-growth factories.

In heuristic search algorithms, performance comes at the cost of being too specific to the problem being solved [32]. By virtue of having few parameters, PSOs are less affected by this problem. In our implementation, we have used parameter values as found in the general PSO literature without the need to adjust them. The only parameters that we adjusted were the number of particles and the number of iterations. We clearly use fewer particles than is typical. This is because we found a population size of 10 to be sufficient for our needs (see Additional file 1: Figure S1). Although we have sampled the entire solution space, particles can easily be forced to explore a subspace. Certain reactions can be excluded from being considered for knockouts by forcing their corresponding indicator variables in the dual system to be 0. Our fitness function is specific to our target design, however new fitness functions can be thought of depending on the desired final objective. Our method produces cMCS leading to designs with similar characteristics as the one used in [24]. Our method also returns multiple solutions. The limiting factor in our method is the MILP for the dual system.

MILPs are more difficult to solve than LPs and may consume large amounts of time as well as memory [33]. During our runs, the search tree generated by CPLEX’s Branch and Cut algorithm for a single MILP grew to consume over 130 GB of memory when limited to a knockout size of 6. This memory consumption grows quickly with increasing knockout size, thereby limiting the ability of PSOMCS to find the optimal solution.

Improvements in run time can be made by forcing PSOMCS to explore only a part of the flux space leading to a smaller solution space to be explored. For instance lets consider the design in Fig. 5, with a minimal biomass yield of 0.01, the optimal design presented here was found within 24 hours. Further improvements to performance could be obtained by following the strategies outlined in [34]. Also, algorithmic improvements in solving MILPs could be useful in this regard.

Here we have dealt only with knockout strategies to design better strains. It can easily be extended to include the concept of regulatory MCS introduced in [9] which combine reaction up/downregulation with knockouts. There are other constraint based methods dealing with intervention strategies like gene knock-ins and up/downregulation. PSOs and swarm intelligence algorithms in general may be used to compliment these methods.

## Conclusion

PSOMCS finds the best possible design in metabolic networks given multiple objectives with the corresponding cMCS. We have demonstrated its capability in finding optimal knockouts and designs in genome-scale metabolic networks. It finds competitive designs compared to standard tools and is orders of magnitude faster than EFM based tools in finding the optimal solution. PSOMCS could be used to predict minimal knockouts resulting in optimal yields in industrially important microorganisms. As the size and quality of metabolic models increase, methods like the one presented here will be even more useful.

## Additional file

Additional file 1
**Figure S1.** Comparison of runtimes for different swarm sizes. (PDF 13.9 kb)

## Abbreviations

APM: Automatic partitioning method
Bio: Biomass
cMCS: Constrained minimal cut set
CPU: Central processing unit
EFM: Elementary flux mode
FBA: Flux balance analysis
FVA: Flux variability analysis
GA: Genetic algorithm
GAMCS: MCS software package based on a GA Irrev: Irreversible
LP: Linear program
MCS: Minimal cut set
MILP: Mixed integer linear programming
P: Product
PSO: Particle swarm optimization
PSOMCS: MCS software package based on PSO
RAM: Random-access memory
Rev: Reversible
S: Substrate
P: Product
