# Supplementary material for: Optimal knockout strategies in genome-scale metabolic networks using particle swarm optimization
Source: BMC Bioinformatics. 2017 Feb 1;18:78. doi: 10.1186/s12859-017-1483-5 (PMC5286819; doi:10.1186/s12859-017-1483-5)
Supplement: Additional file 1 — Figure S1. Comparison of runtimes for different swarm sizes. (PDF 13.9 kb) [file 12859_2017_1483_MOESM1_ESM.pdf]

SUPPLEMENTARY DATA

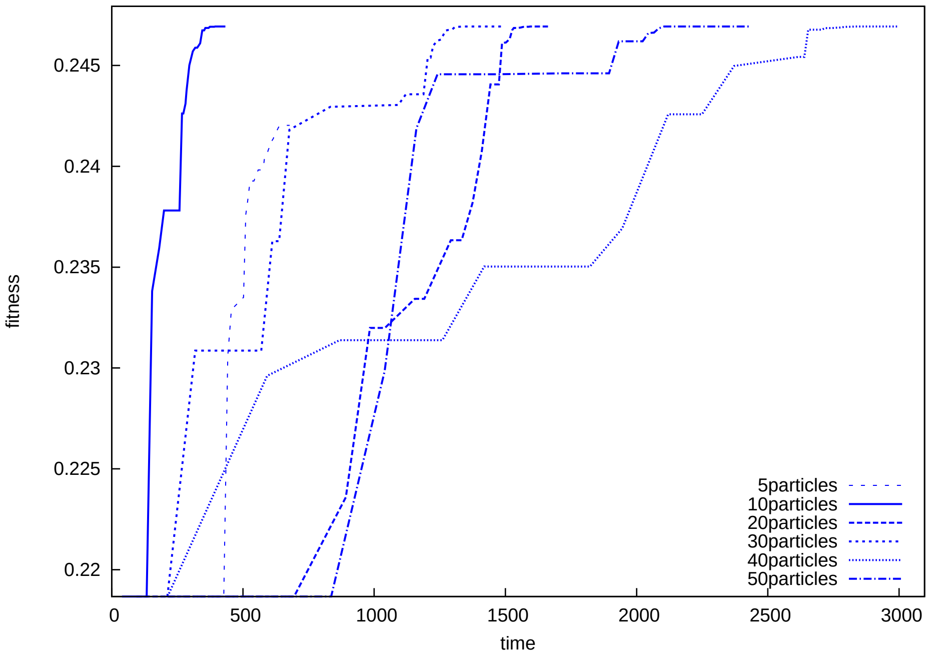

Figure S 1: **Comparison of runtimes for different swarm sizes.** The time taken for swarms of different sizes to reach the maximum fitness in the *E. coli* core model is plotted. It is clear that a size of 10 is faster than larger swarms and a smaller size of 5 fails to reach the optimum.
